# Clustering of Health-Related Behavior Patterns and Demographics. Results From the Population-Based KORA S4/F4 Cohort Study

**DOI:** 10.3389/fpubh.2018.00387

**Published:** 2019-01-22

**Authors:** Matthias Rabel, Michael Laxy, Barbara Thorand, Annette Peters, Lars Schwettmann, Filip Mess

**Affiliations:** ^1^Department of Sport and Health Sciences, Technical University of Munich, Munich, Germany; ^2^Helmholtz Zentrum München–German Research Center for Environmental Health (GmbH), Institute of Health Economics and Health Care Management, Neuherberg, Germany; ^3^German Center for Diabetes Research (DZD), Neuherberg, Germany; ^4^Helmholtz Zentrum München–German Research Center for Environmental Health (GmbH), Institute of Epidemiology, Neuherberg, Germany

**Keywords:** health behavior pattern, latent class analysis, latent class regression, cluster analysis, alcohol, nutrition, physical activity, smoking

## Abstract

**Background:** Health behaviors are of great importance for public health. Previous research shows that health behaviors are clustered and do not occur by chance. The main objective of this study was to investigate and describe the clustering of alcohol consumption, nutrition, physical activity and smoking while also considering the influence of sex, age and education.

**Methods:** Using data from the population-based KORA S4/F4 cohort study, latent class regression analysis was undertaken to identify different clusters of health behavior patterns. The clusters were described according to demographics. Furthermore, the clusters were described regarding health-related quality of life at baseline and at a 7 year follow-up.

**Results:** Based on a sample of 4,238 participants, three distinct classes were identified. One overall healthy class and two heterogeneous classes. Classes varied especially according to sex, indicating a healthier behavior pattern for females. No clear association between healthier classes and age, education or physical and mental health-related quality of life was found.

**Discussion:** This study strengthens the literature on the clustering of health behaviors and additionally describes the identified clusters in association with health-related quality of life. More research on associations between clustering of health behaviors and important clinical outcomes is needed.

## Introduction

Health behaviors are closely linked to a person's general health. This is not only postulated in theoretical models like the determinants of health-model (1), but has also been shown empirically (2). In addition to the individual level, health behaviors also have large public health implications. Particularly, alcohol consumption, nutrition, physical activity (PA) and smoking are well-known and important factors regarding public health. In its report on global health risks, the World Health Organization (WHO) lists health behaviors among the leading risk factors for death in high-income countries (3, 4). Plenty of studies have investigated the association between one of these single risk behaviors and health (5–9). However, health-impairing factors usually do not occur apart, but tend to group in clusters (10, 11). A bundling of different health risks can be identified (12) and certain population strata show common patterns of multiple health behaviors (13–15). As there is evidence that multiple health behaviors have synergistic effects and might be targeted simultaneously by interventions, analyzing patterns of health behaviors can be of great importance for public health (16). First, there is evidence that interventions which tackle multiple behaviors seem to be more cost effective (17). Second, with the already described bundling of behaviors and a common culmination of risk factors for individuals (12), considering more than one health behavior to describe individual health patterns seems to be appropriate. Third, in order to design health promoting interventions it is crucial to identify the characteristics of the target group (18), which requires an understanding of how multiple health behaviors are clustered (19). In-depth knowledge about the characteristics of a population might be helpful to identify vulnerable groups, which could profit most from public health interventions (20).

Multiple studies have investigated the general clustering of health behaviors in adults (11, 15, 20, 21). Yet, due to methodological differences (22) as well as different investigated health behaviors (11), the literature is quite heterogeneous. Furthermore, only few studies have investigated the associations between the general clustering of health behaviors and important physical health outcomes like all-cause mortality (7, 23–25).

Health-related quality of life (HRQOL) is an important patient relevant outcome combining physical and mental aspects of health (26). Due to the rise of chronic diseases the concept of HRQOL is crucial for public health (27). Several studies have investigated associations between single health behaviors and HRQOL (28–31). However, little is known about the relationship between patterns of several health behaviors and HRQOL at the population level. Dumuid et al. (32) identified four distinct clusters based on PA, nutrition and screen time in school-aged children. They report differences in HRQOL scores with highest scores for a mixed cluster (low screen time, healthy eating, and moderate PA) (32). Another study investigated associations between a clustering of healthy behaviors (non-smoking, adequate PA, consumption of at least five portions of fruit or vegetables per day) and HRQOL in US adults with diabetes. According to this study, an increase of healthy behaviors is associated with better HRQOL (33). To the best of our knowledge, only one study investigated the relationship of the clustering of health behaviors and HRQOL in a general population (16). Based on smoking, drinking alcohol, PA and nutrition, six health behavior clusters were identified. The scientists report that healthier clusters tend to be associated with better aspects of HRQOL (16). To the best of our knowledge, no study investigated the association between the clustering of health behaviors and HRQOL, including multiple measurements of HRQOL at different time points. Due to this reason, the present study uses data from a population-based cohort study including information on HRQOL at two time points approximately 7 years apart.

The main objective of this study was the identification of clusters that share a similar pattern based on their health behavior. It is assumed that health behavior patterns are closely linked to demographic factors (34). Therefore, sex, age and education are also included in the clustering process. The considered behaviors are smoking, alcohol consumption, leisure time PA, and nutrition. In order to attain comparability with other clustering solutions and with regard to the public health impact of these four behaviors, the present study considers these behaviors in order to identify health behavioral clusters. In order to receive a profound understanding of the identified clusters and characterize their differences, it is helpful to describe them with additional parameters (35). Therefore, the identified clusters will be described regarding sociodemographic parameters. With respect to the sparse scientific background on the association between the clustering of health behaviors and HRQOL, the clusters will be additionally described regarding longitudinal change in physical and mental HRQOL between baseline and a 7-year follow-up.

## Methods

### Data Source

Our analyses are based on data obtained from the population-based KORA (Cooperative Health Research in the Region of Augsburg) S4/F4 cohort study. In total 4,261 non-institutionalized inhabitants of the Augsburg Region in Southern Germany took part in the baseline health survey conducted in 1999-2001 (S4). All S4 participants were invited to participate in a follow-up examination approximately 7 years later (F4; 2006-2008). A total of 3,080 (72%) participants were investigated at follow-up. The reasons for losses at follow-up are as follows: death (176), claim for data deletion (12), no contact possible (174), refusal to participate (395), illness/lack of time (218), no contact information available (206). At baseline and at follow-up a physical examination and standardized interviews were performed. More detailed information on the KORA S4 study concerning sampling methods and data collection has been published elsewhere (36). All study participants gave written informed consent and the KORA S4/F4 studies were approved by the Ethics Committee of the Bavarian Medical Association.

### Measures

#### Alcohol

Alcohol consumption was calculated based on participants' self-reported information on beer, wine and spirits consumption on the previous workday and weekend. The weekday consumption was multiplied by five and added to the weekend consumption. Total alcohol intake per day [g/day] was derived from dividing this number by seven. Details on the calculation and validation process have already been published (37). For the present study alcohol consumption has been grouped into the following three categories: (1) 0 g/day = “no alcohol,” (2) < 20 g/day = “moderate consumption,” (3) ≥20 g/day = “risky consumption” as suggested by published classifications (38).

#### Nutrition

Dietary intake was collected by using a food-frequency-questionnaire investigating 24 food groups. Based on recommendations of the German Nutrition Society (DGE) an index was built rating the frequency with which each food was consumed by assigning either 2, 1, or 0 points. Higher scores reflect better compliance to DGE recommendations. A resulting sum score was then rated according to DGE guidelines grouped into the following three categories: (1) “favorable,” (2) “ordinary,” and (3) “adverse.” This approach was established in earlier KORA studies and was validated against a detailed seven-days-dietary protocol (39).

#### Physical Activity

Participants were asked to report their weekly time spent on leisure-time PA (including cycling) in summer and winter. The two responses were combined and categorized into four groups: (1) “(almost) no activity,” (2) “about 1 h per week irregularly,” (3) “about 1 hour per week regularly,” and (4) “regularly more than 2 h per week.” Categories (2) and (3) were condensed for all statistical analyses because they were not considered adequately distinct. The questions about leisure-time PA originated from the German Cardiovascular Prevention Study conducted between 1979 and 1995 and were validated in the KORA population, by using a PA diary (40).

#### Smoking

Smoking behavior was classified considering the actual behavior and the regularity with which the behavior was performed. Therefore, participants were asked whether they currently smoke and whether they have ever smoked. Additionally, regularity was surveyed by inquiring whether someone usually smokes regularly (at least one cigarette per day) or irregularly (usually less than one cigarette per day). According to this information, smoking behavior was grouped into (1) non-smoker, (2) ex-smoker, (3) irregular smoker, and (4) regular smoker.

#### Health-Related Quality of Life

HRQOL was estimated with the SF-12 health survey. This short version of the SF-36 health survey consists of 12 items distinguishing between physical and mental components of HRQOL. Both components can be summarized in a physical component summary score and a mental component summary score with a mean of 50 points and a standard deviation of ten points. Higher scores imply a better HRQOL. Information about psychometric quality criteria can be found elsewhere (41).

#### Covariates

Information about sex (male, female), age (continuous) and education (main-, middle- and grammar school corresponding to German “Hauptschule,” “Realschule,” and “Gymnasium”) were also collected during the standardized interviews.

### Statistical Analysis

In order to identify homogeneous subgroups based on the samples' health behaviors, a latent class analysis was carried out. This method allows to cluster cases into unobserved classes. An advantage of latent class analysis compared to traditional clustering techniques like hierarchical clustering or k-means is that it is a model-based approach. Therefore, this approach is more flexible and the choice of a cluster criterion is less arbitrary (42). Key assumptions to this approach are that the latent class variable and the observed variables, in this case the health behaviors alcohol consumption, nutrition, PA and smoking, are treated as categorical. Another assumption is that the observed variables are locally independent and that dependencies are conditional on the latent classes' variable (43). In a basic, unconditional model, latent class models analyze cross-classification tables of observed variables to estimate the probability of latent class membership. This unconditional model can be extended with covariates which influence latent class membership, into a latent class regression analysis. While in the unconditional model it is assumed that every individual has the same probability for class membership, this approach allows for varying probabilities depending upon observed covariates (44). In our study, sex, age, and education were added to the analysis as covariates, since their importance in this process has been stressed in past studies (11). Latent class membership is based solely on data from the S4 study (1999-2001). Figure A1 in the Appendix gives a visual overview of the latent class regression model. Since in this case, the number of latent classes is unknown, multiple models with an increasing number of classes were fit. To identify the most parsimonious model that represents the data best, several model fit criteria including Akaike information criterion (AIC) and (sample-sized-adjusted) Bayesian Information Criterion (BIC) were deployed. In this study we prioritized BIC as it is the most commonly used criterion which is supposed to be very accurate (45). In addition, model selection should also consider the practical meaning of identified classes (46) and therefore a complex model that cannot provide a new meaningful class is seen as inferior to a simpler model with a marginally worse model fit. Cluster analysis is an analysis to identify groups/clusters. In latent class analysis the term for these groups is class. Therefore, the terms will be used synonymously in this paper.

Descriptive statistics were used to describe and interpret the different latent classes. To investigate differences in HRQOL at baseline (S4) and the follow-up (F4) and to investigate the change in HRQOL between the measurement points a mixed model with a random intercept was fit. In this model HRQOL is the dependent variable and latent class membership, and an interaction between latent class membership and measurement time point are independent variables. Sex, age, and education were introduced as covariates in the model. Altogether two mixed models were fit to differentiate between physical and mental HRQOL.

All data related processes and statistical analyses were conducted using R (Version 3.4.3) (47). Latent class regression analysis was carried out using the “poLCA” package (Version 1.4.1) (44).

## Results

### Participants

**Figure 1:**
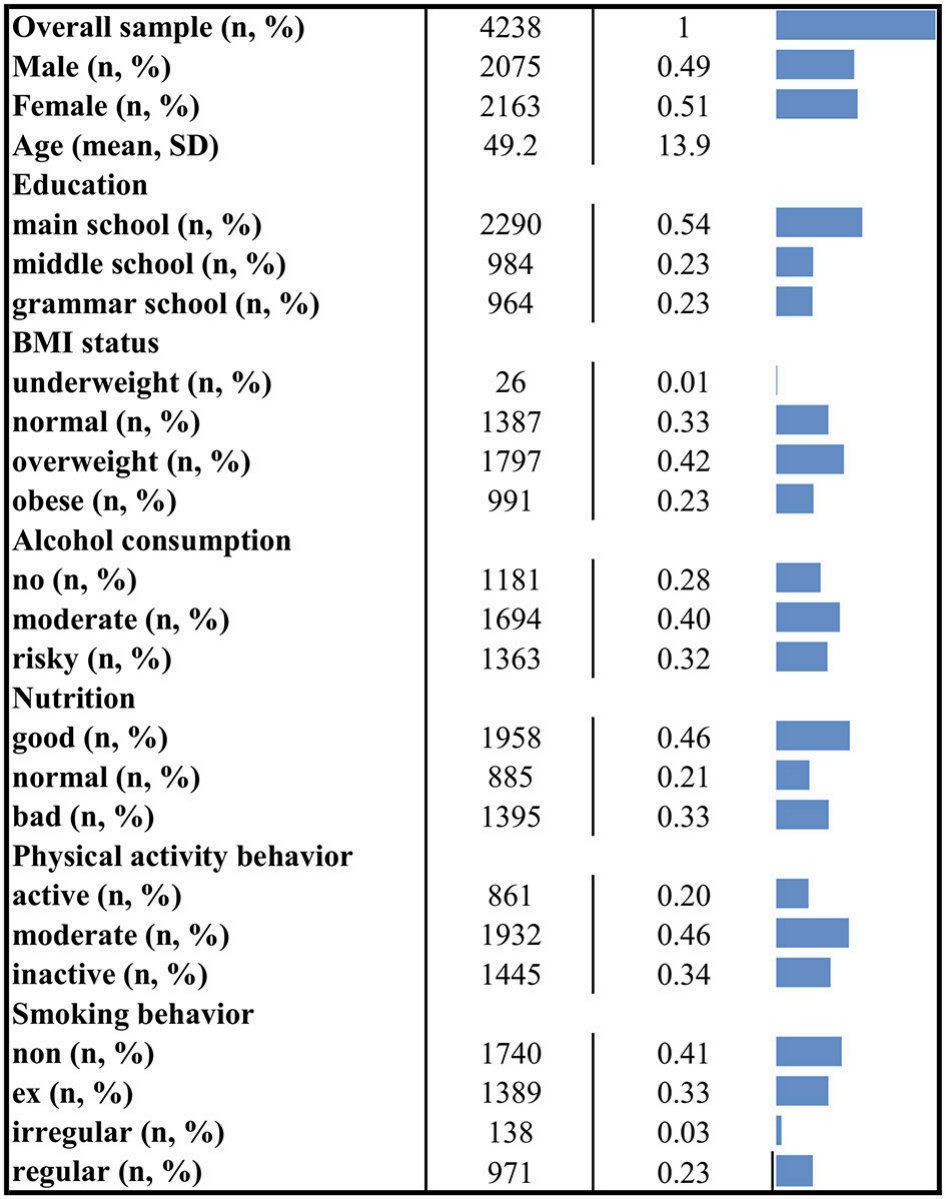
Socio-demographic characteristics and distribution of health behaviors. The figure displays descriptive information for the overall applicable sample. N, number of observations; %, column percent (except for the first row); SD, standard deviation.

Twenty three participants (0.5%) of the baseline S4 study were excluded from the latent class regression analysis due to missing data in the behavioral variables or the covariates. The remaining sample of 4,238 had a mean age of 49.2 (±13.9) years ranging from 24 to 75 years. 49% of the sample were male, 54% graduated from main school, 23.2% from middle school and 22.7% from grammar school. One third of the population had a normal BMI (≥18.5– <25 kg/m^2^). Nearly two third of the participants were overweight (BMI≥25– <30 kg/m^2^) or obese (BMI≥30 kg/m^2^). Only 0.6% was underweight (BMI <18.5 kg/m^2^). Figure 1 shows more socio-demographic details and the distribution of the health behavior categories for the overall sample.

### Class Selection

**Figure 2:**
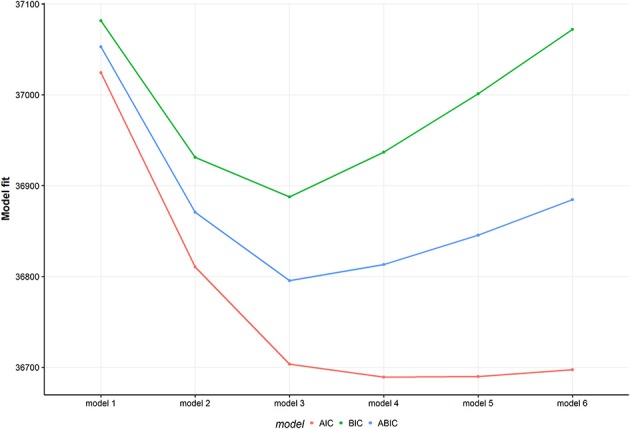
Model fit for multiple models. The figure displays three different model fit criteria for different models. Smaller values indicate a better model fit. The model number represents the number of latent classes. AIC, Akaike Information Criterion; BIC, Bayesian Information Criterion; ABIC, sample size adjusted BIC.

**Figure 3:**
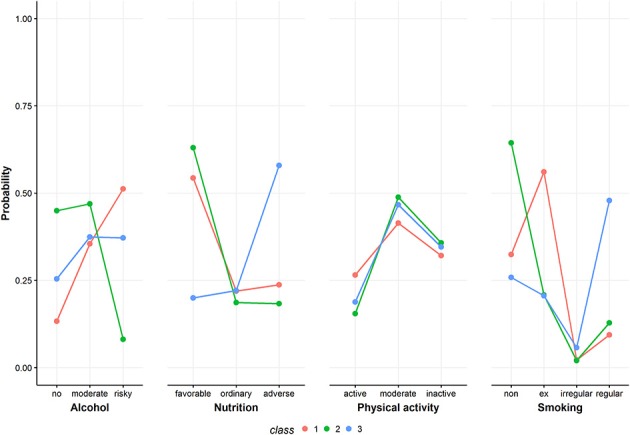
Class-conditional item probabilities. The figure shows the probabilities of each health behavior category conditional on latent class membership.

Figure 2 shows model fit criteria for the different models. Smaller y-values indicate a better model fit. Multiple models with an increasing number of classes from one to six were fit. The two-class (BIC = 36931.16), three-class (BIC = 36887.63) and four-class (BIC = 36936.96) solutions showed the best model fit regarding the BIC criteria. After a substantial comparison of all solutions, the four-class solution did not reveal any new unique behavioral pattern. Therefore, the three-class solution was chosen as the most appropriate model. For comparison, see Figure 3 and Figures A2, A3 in the Appendix showing the class-conditional item probabilities.

### Description of the Identified Groups

Figure 3 shows the class-conditional item probabilities. This plot displays the probability of an item response given latent class membership. The specific values are presented in Table A1 in the Appendix.

**Figure 4:**
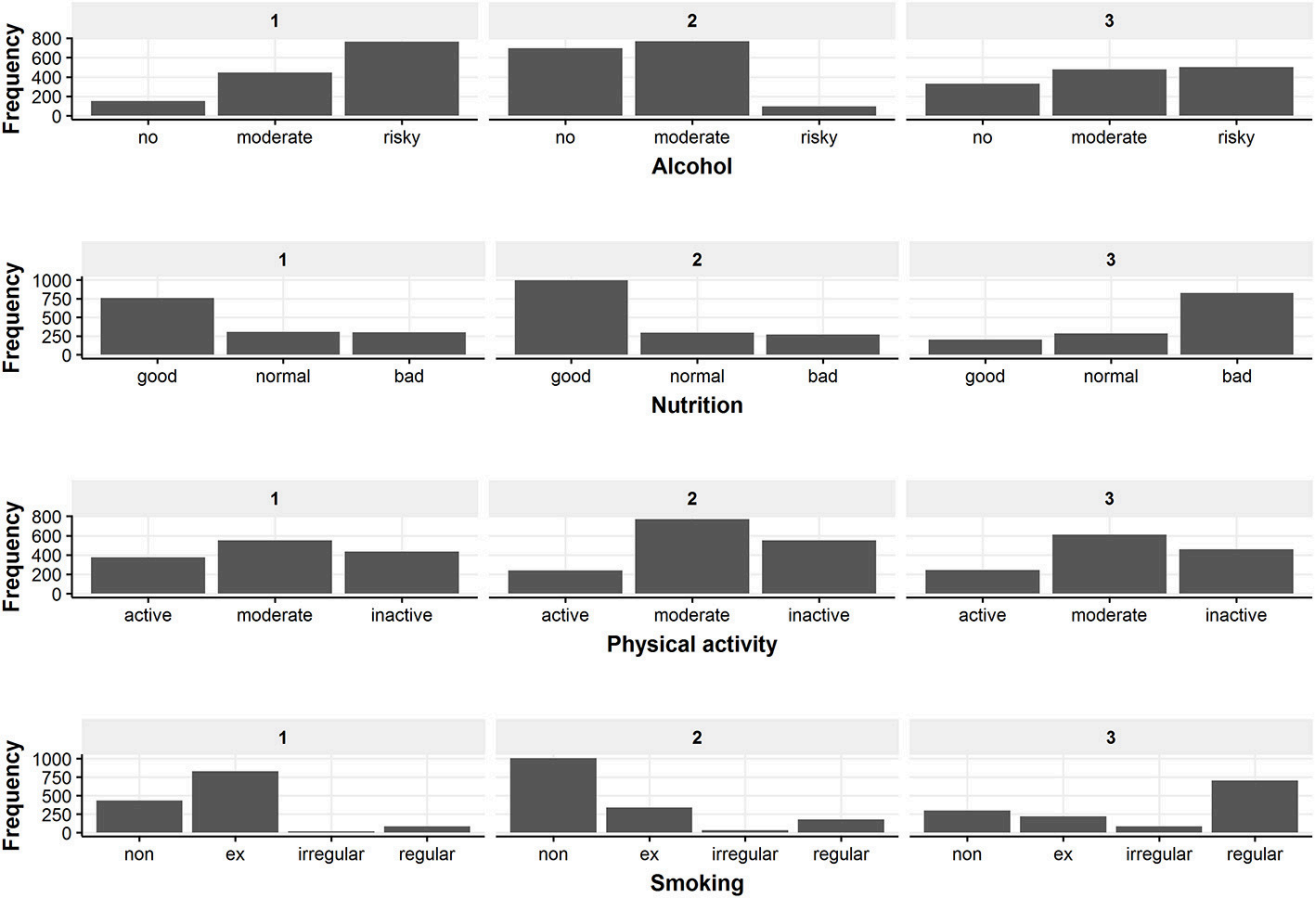
Overview of the behavior distribution in the three latent classes. The figure displays the frequency of every health behavior category for the three classes.

**Figure 5:**
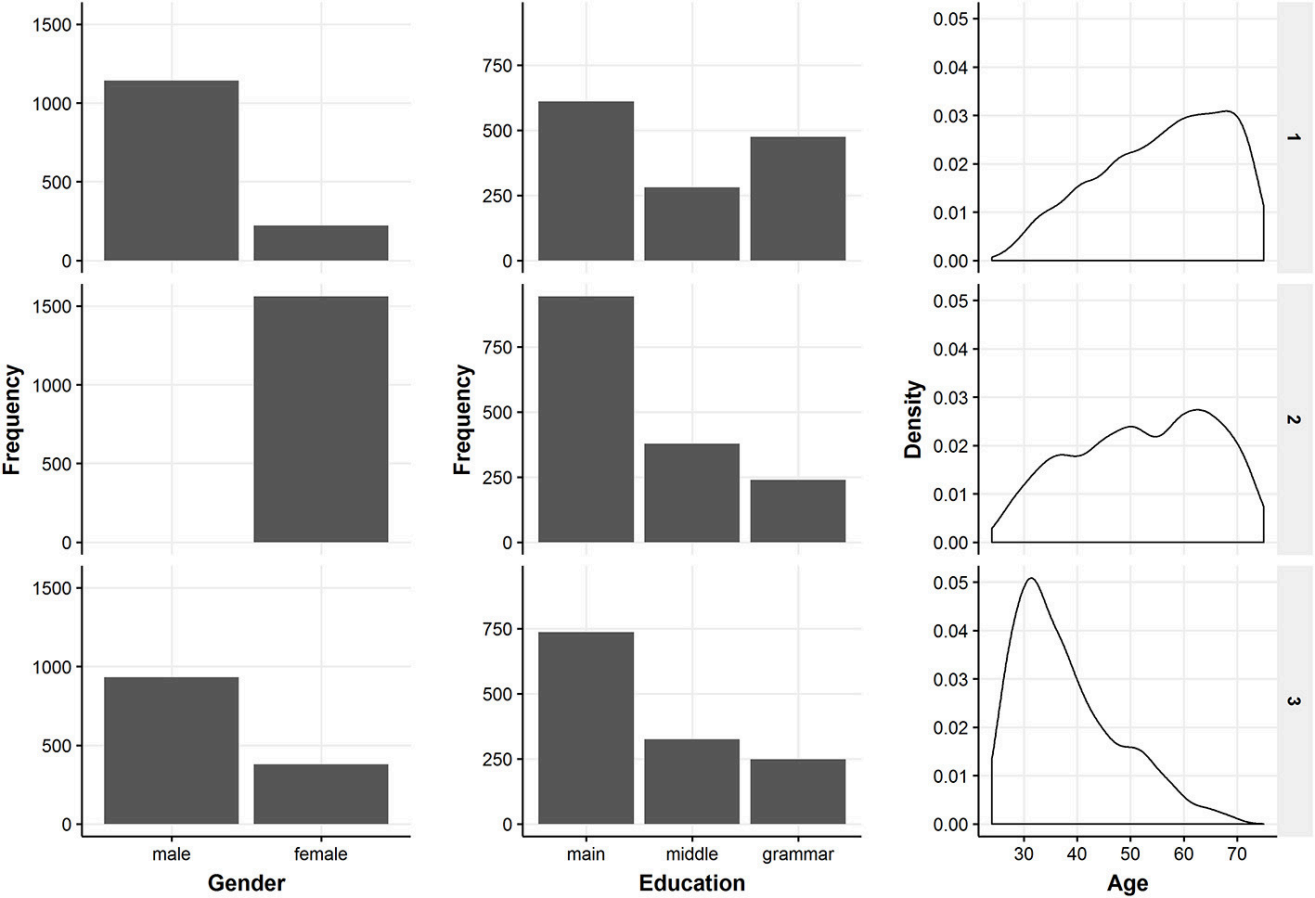
Overview of the included covariates in the three latent classes. The figure shows descriptive information on gender, education and age which were included in the latent class regression.

In the following, the three classes from the selected three-class model solution are described regarding their health behavior patterns and socio-demographic characteristics. Figure 4 gives a detailed overview of the health behaviors of the identified classes. Information on the distribution of the three covariates included in the latent class regression can be found in Figure 5. Exact numbers on the distribution of the health behaviors and the covariates can be seen in Table A2 in the Appendix.

#### Class 1

Class 1 has 1,366 members of which 84% are male. Mean age is 56.1 (±11.9) years, 45% are main school attendees and it is the class with the most grammar school graduates (35%). 0.1% are underweight, 26% are of normal weight, 50% are overweight and 24% are obese. Conditional on class 1 membership, class members have the highest probability for risky drinking (51%), a favorable diet (54%), moderate PA (41%) and ex-smoking (56%).

#### Class 2

Class 2 is the biggest class. Class 2 consists of 1,562 participants and is exclusively female. The biggest share are main school attendees (60%) and the mean age is 52.4 (± 13.0) years. 0.5% are underweight, 34% have a normal weight, 37% are overweight and 29% are obese. Regarding health behavior, class 2 membership has the highest probability for drinking no (45%) or moderately (47%) alcohol, having a favorable diet (63%), being moderately physically active (49%), and being non-smoking (64%).

#### Class 3

Class 3 is composed of 1,310 participants, 71% thereof are male. Most participants of class 3 received a main school educational degree (56%). Class 3 is the youngest class with a mean age of 38.1 (± 9.8) years. Most participants have normal weight (40%) or are overweight (43%). 1.3% are underweight and 16% are obese. Concerning health behavior, class 3 membership implies highest probabilities for moderate (37%) or risky (38%) alcohol consumption, adverse dietary behavior (58%), moderate PA (47%) and regular smoking (48%).

### Associations Between Latent Classes and Health-Related Quality of Life

Based on the mixed model, class 1 (mean = 48.88; 95%-CI = 48.36–49.41) and 2 (mean = 48.47, 95%-CI = 47.89–49.06) have the highest physical HRQOL at baseline. Class 3 has a mean score of 46.96 (95%-CI = 46.28–47.55) concerning physical HRQOL. At follow-up, mean values for physical HRQOL are 47.64 (95%-CI = 47.07–48.21) for class 1, 47.62 (95%-CI = 47.00–48.25) for class 2 and 47.17 (95%-CI = 46.51–47.82) for class 3.

Regarding mental HRQOL, class 1 had a mean score of 51.06 (95%-CI = 51.37–52.17), class 2 had a mean score of 50.77 (95%-CI = 50.14–51.40) and class 3 had a mean score of 50.39 (95%-CI = 49.76–51.02). Follow-up values for mental HRQOL were 51.32 (95%-CI = 50.70–51.95) for class 1, 50.95 (95%-CI = 50.27–51.63) for class 2 and 51.18 (95%-CI = 50.47–51.89) for class 3.

**Figure 6:**
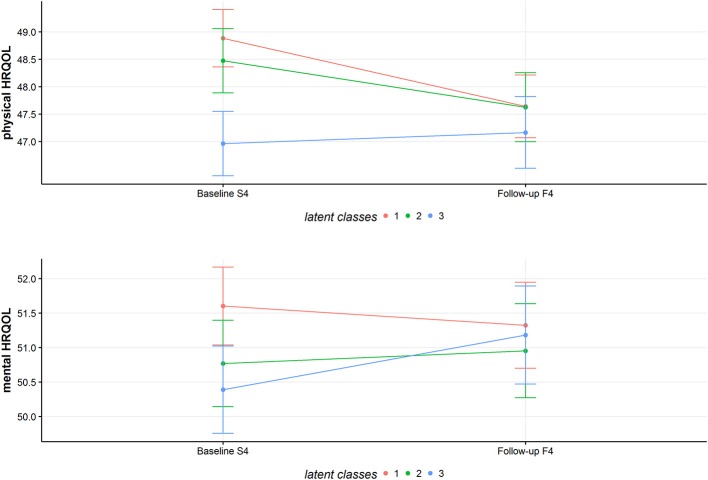
Predicted mean values of physical and mental HRQOL for the latent classes. The figure shows the estimated marginal means for physical and mental HRQOL for the three classes at baseline and follow-up.

Figure 6 provides a visual overview of the mean physical and mental HRQOL of the three classes.

## Discussion

This study aimed at identifying different subgroups of a population based on the health behaviors alcohol consumption, nutrition, PA and smoking, while also taking the influence of the parameters sex, age and education into account. Three distinct classes were identified. All three classes show a unique pattern regarding the health behaviors. Class 2 represents a healthy cluster, showing a very healthy pattern and the highest item probabilities for healthy behavior categories. Class 1 and class 3 on the other hand show more unhealthy profiles. Class 1 shows highest item probabilities for a risky alcohol consumption. In addition, class 1 has the highest probability for former smoking which has been associated with higher odds for hospital treatments and higher numbers in physician visits (48).

Cluster analysis is very exploratory and although comparisons with other studies are difficult because of different investigated health behaviors and methodological approaches, our results are in line with similar investigations. In line with previous studies, we identified an overall healthy cluster with class 2 (13, 14, 16, 20, 21, 49, 50). Similar to previous studies, we observed a clustering of unhealthy smoking behavior and unhealthy alcohol consumption (11, 15, 16, 20). This clustering becomes very evident for class 3 and partially for class 1 considering the high number of ex-smokers in this class. Some studies report a combination of excessive alcohol consumption and higher PA rates (14, 16, 49). A tendency of this combination could be observed in class 1, which has highest item probabilities for drinking and the highest probability for active PA amongst the three classes.

Regarding socio-demographic characteristics of the identified clusters, the present results are in line with previous studies. Previous research reported a higher male prevalence in more unhealthy clusters (14, 20). Our findings support this result, showing the highest female prevalence rates for the healthy class 2. In our study, age and education were not a good indicator for distinguishing between healthy and unhealthy clusters, as younger and older clusters or more and less educated clusters can be found on both ends of the spectrum. A similar result is also mentioned in the systematic review of Meader et al. (11).

Scientific evidence on associations between clustering of health behaviors and HRQOL is sparse. Conry et al. (16) report a tendency for healthier clusters having a better quality of life. This result could not be replicated by our study. We found no clear association between a healthier behavior pattern and better physical or mental HRQOL. The different latent classes show different adjusted means in physical and mental HRQOL. The classes are also different in their change of physical or mental HRQOL over the years. However, the different changes in physical/mental HRQOL might be due to regression to the mean. Furthermore, the differences in physical/mental HRQOL are too small to be considered as clinically relevant. For the SF-12 questionnaire a difference from three to five points can be seen as the minimal clinically relevant threshold (51).

Our study has several limitations. One problem lies in the way the health behaviors are measured and operationalized. All information on health behaviors is self-reported and thus prone to information bias like recall-bias or social desirability-bias. Furthermore, taking average scores for PA by combining information on winter and summer can be considered as another weakness. However, especially in large cohort studies with many variables, one has to balance the tradeoff between accuracy and feasibility. Another limitation lies in the cross-sectional design of this study. The clustering of the health behaviors is based on the baseline-study and therefore can only be seen as a snapshot. Nevertheless, the questions in the KORA-study are conceptualized to gather information on an established behavior. A further drawback is the assumption that the health behavior pattern which was identified at baseline, remains stable over time. Even though, this assumption might be very bold, this is an adult population and there is evidence, that health behavior patterns are quite stable over time (52, 53). Another limitation of this study concerns the longitudinal description of HRQOL for the established clusters at baseline. The reduced sample size of the KORA-study might result in a biased depiction of HRQOL as healthier people with better HRQOL are more likely to remain in the study. Taking this into account, the observed changes in physical and mental HRQOL might not necessarily reveal a true change on a population level. Although we adjusted our analyses for several variables, chances are high that HRQOL might have been influenced by a factor we did not adjust for, e.g., socio-economic status. Therefore, residual confounding cannot be ruled out.

Besides the aforementioned limitations, this study has several noticeable strengths. The data are collected in a large population-based cohort study. The clustering is based on a latent class model. This approach offers a clustering based on a statistical model instead of more arbitrary cluster criteria and thus might be more sophisticated than traditional clustering approaches (54). Moreover, this methodological approach allows introducing covariates to factor in their influence on health behavior patterns. Another strength of this study is that the clustering is not only based on dichotomous variables like the absence or presence of a risk factor but also on polytomous variables. Despite its limitations and the therefore limited level of inference, addressing the relevance of health behavior clusters by linking them to HRQOL—a clinically important outcome—adds value to this study.

In conclusion, this study identified distinct patterns of health behaviors within a large population-based sample. The observed health behavior patterns and the socio-demographic characteristics of the identified clusters are in line with the few other existing international studies. Knowledge on specific clusters which are common in an adult population are an important step for comprehensive health promoting public health policies. The clustering of lifestyle factors like health behaviors can give valuable information on characteristics of target groups for primary preventions. Combining these findings with further information on big data by health care providers or individual risk probabilities might result in even more effective and comprehensive health care. Further research on health behavior patterns should focus on linking identified clusters to important medical outcomes in order to identify vulnerable groups and to allow for individualized patient-centered primary prevention programs.

## Author Contributions

MR, FM, and ML: Conceptualization. MR: Formal analysis, investigation, methodology, and writing-original draft. BT, AP, LS, ML, and FM: Supervision. MR, BT, AP, LS, ML, and FM: Writing–review and editing.

### Conflict of Interest Statement

The authors declare that the research was conducted in the absence of any commercial or financial relationships that could be construed as a potential conflict of interest.
