# Supplementary material for: Clustering of Health-Related Behavior Patterns and Demographics. Results From the Population-Based KORA S4/F4 Cohort Study
Source: Front Public Health. 2019 Jan 22;6:387. doi: 10.3389/fpubh.2018.00387 (PMC6350271; doi:10.3389/fpubh.2018.00387)
Supplement: Supplementary file 1 [file Data_Sheet_1.pdf]

Appendix

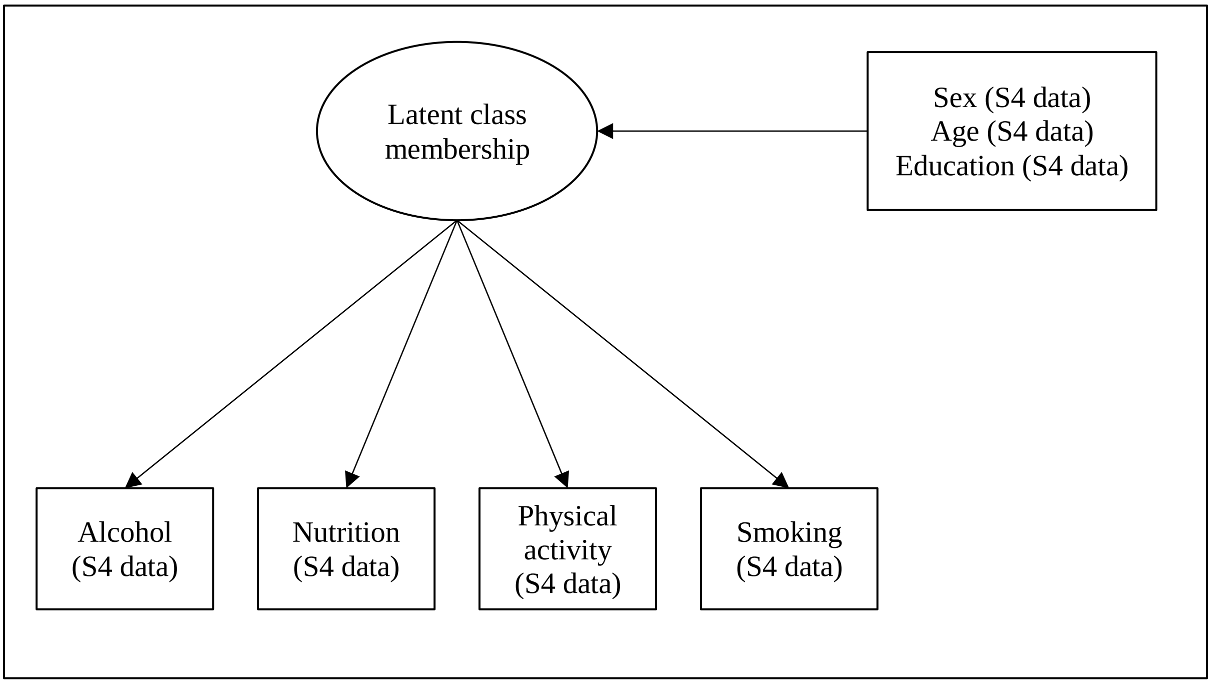

Figure A1. The latent class regression model.
A graphical description of the latent class regression model based on baseline (S4) data.

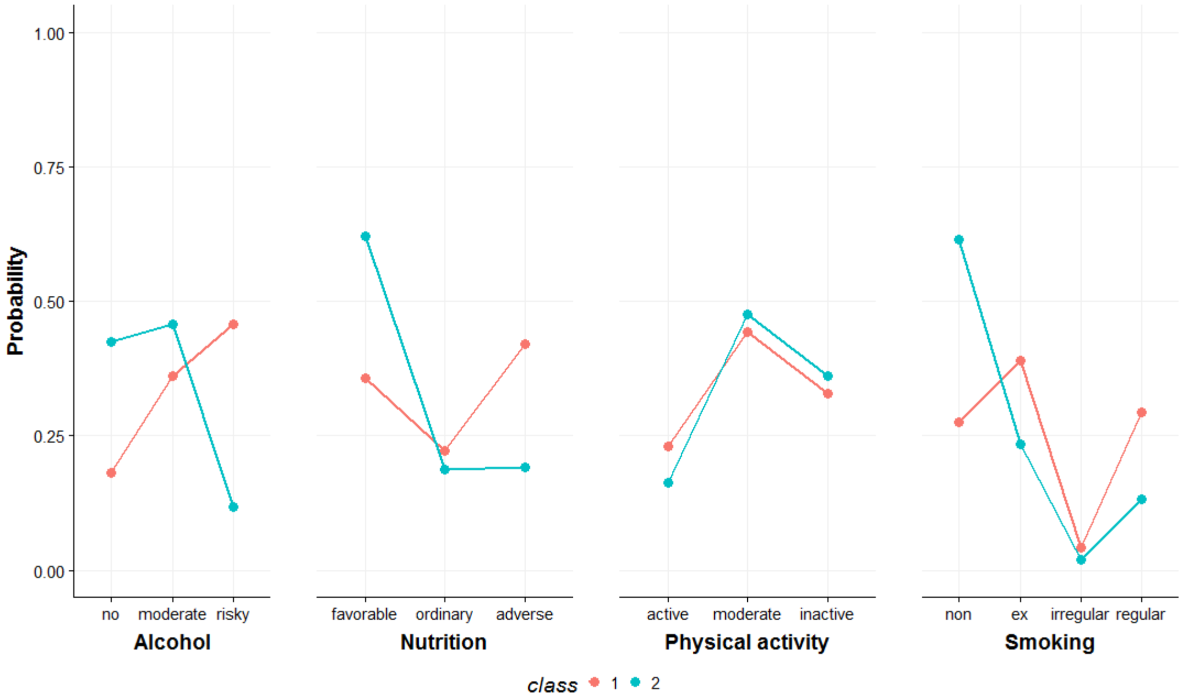

Figure A2. Two-class-conditional item probabilities.
The figure shows the probabilities of each health behavior category conditional on latent class membership for a two-class model.

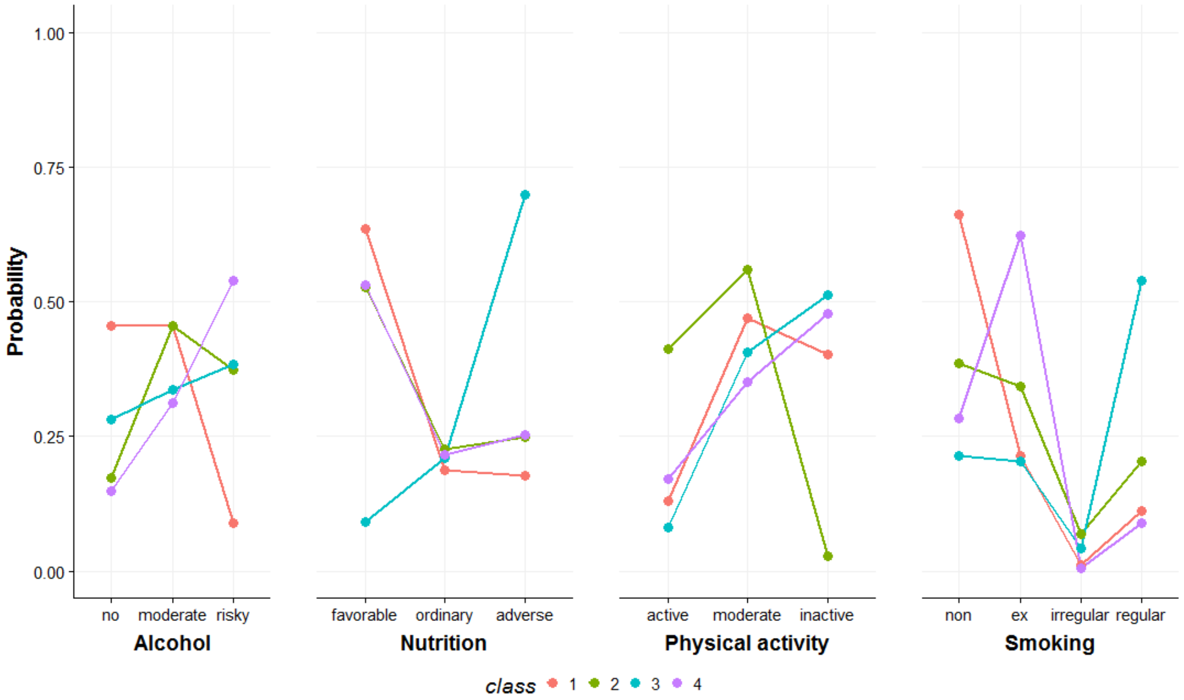

Figure A3. Four-class-conditional item probabilities.
The figure shows the probabilities of each health behavior category conditional on latent class membership for a three-class model.

Table A1. Three-class-conditional item probabilities exact values
Referring to Figure 3 in the manuscript, this table shows the exact numerical values of the class-conditional item probabilities.

		Class 1	Class 2	Class 3
**Alcohol**	**no**	0.133	0.450	0.254
	**moderate**	0.355	0.469	0.374
	**risky**	0.512	0.081	0.372
**Nutrition**	**favorable**	0.543	0.630	0.200
	**ordinary**	0.219	0.187	0.221
	**adverse**	0.237	0.183	0.579
**Physical activity**	**active**	0.265	0.155	0.188
	**moderate**	0.414	0.488	0.466
	**inactive**	0.321	0.357	0.346
**Smoking**	**non**	0.324	0.644	0.258
	**ex**	0.561	0.208	0.206
	**irregular**	0.022	0.020	0.057
	**regular**	0.094	0.128	0.479

Table A2. Further descriptive statistics on latent classes.
In addition to the reported information in the manuscript, this table shows further descriptive information concerning the three latent classes.

	Latent Classes		
	**I**	**II**	**III**
**Overall sample (n, %)**	1366 (32.2)	1562 (36.9)	1310 (30.9)
**Male (n, %)**	1143 (83.7)	0 (0.0)	932 (71.1)
**Female (n, %)**	223 (16.3)	1562 (100.0)	378 (28.9)
**Age (mean, SD)**	56.1 (11.9)	52.4 (13.0)	38.1 (9.8)
**Education**			
**main school (n, %)**	610 (44.7)	944 (60.4)	736 (56.2)
**middle school (n, %)**	281 (20.6)	378 (24.2)	325 (24.8)
**grammar school (n, %)**	475 (34.8)	240 (15.4)	249 (19.0)
**Alcohol consumption**			
**no (n, %)**	153 (11.2)	697 (44.6)	331 (25.3)
**moderate (n, %)**	447 (32.7)	769 (49.2)	478 (36.5)
**risky (n, %)**	766 (56.1)	96 (6.1)	501 (38.2)
**Nutrition**			
**good (n, %)**	761 (55.7)	997 (63.8)	200 (15.3)
**normal (n, %)**	306 (22.4)	297 (19.0)	282 (21.5)
**bad (n, %)**	299 (21.9)	268 (17.2)	828 (63.2)
**Physical activity behavior**			
**active (n, %)**	377 (27.6)	241 (15.4)	243 (18.5)

**moderate (n, %)**	552 (40.4)	771 (49.4)	609 (46.5)
**inactive (n, %)**	437 (32.0)	550 (35.2)	458 (35.0)
**Smoking behavior**			
**non (n, %)**	432 (31.6)	1009 (64.6)	299 (22.8)
**ex (n, %)**	830 (60.8)	339 (21.7)	220 (16.8)
**irregular (n, %)**	18 (1.3)	34 (2.2)	86 (6.6)
**regular (n, %)**	86 (6.3)	180 (11.5)	705 (53.8)
